# Sulforaphane Inhibits MGO-AGE-Mediated Neuroinflammation by Suppressing NF-κB, MAPK, and AGE–RAGE Signaling Pathways in Microglial Cells

**DOI:** 10.3390/antiox9090792

**Published:** 2020-08-26

**Authors:** Lalita Subedi, Jae Hyuk Lee, Bhakta Prasad Gaire, Sun Yeou Kim

**Affiliations:** 1 College of Pharmacy, Gachon University, #191, Hambakmoero, Yeonsu-gu, Incheon 21936, Korea; subedilali@gmail.com (L.S.); wogur6378@naver.com (J.H.L.); samarpanbp@gmail.com (B.P.G.); 2 Gachon Institute of Pharmaceutical Science, Gachon University, #191 Hambakmoe-ro, Yeonsu-gu, Incheon 21936, Korea; 3 Gachon Medical Research Institute, Gil Medical Center, Incheon 21565, Korea

**Keywords:** sulforaphane, advanced glycation end products, neuroinflammation, microglial activation

## Abstract

Advanced glycation end products (AGEs) are produced through the binding of glycated protein or lipid with sugar, and they are known to be involved in the pathogenesis of both age-dependent and independent neurological complications. Among dicarbonyl compounds, methylglyoxal (MGO), which is produced from glucose breakdown, is a key precursor of AGE formation and neurotoxicity. Several studies have shown the toxic effects of bovine serum albumin (BSA)-AGE (prepared with glucose, sucrose or fructose) both in in vitro and in vivo. In fact, MGO-derived AGEs (MGO-AGEs) are highly toxic to neurons and other cells of the central nervous system. Therefore, we aimed to investigate the role of MGO-AGEs in microglial activation, a key inflammatory event, or secondary brain damage in neuroinflammatory diseases. Interestingly, we found that sulforaphane (SFN) as a potential candidate to downregulate neuroinflammation induced by MGO-AGEs in BV2 microglial cells. SFN not only inhibited the formation of MGO-AGEs, but it did not show breaking activity on the MGO-mediated AGEs cross-links with protein, indicating that SFN could potentially trap MGO or inhibit toxic AGE damage. In addition, SFN significantly attenuated the production of neuroinflammatory mediators induced by MGO-AGEs in BV2 microglial cells. SFN also lowered the expression levels of AGE receptor (RAGE) in microglial cells, suggesting that SFN could downregulate MGO-AGE-mediated neurotoxicity at the receptor activation level. Altogether, our current study revealed that SFN might show neuropharmacological potential for downregulating MGO-AGEs-mediated neuronal complications thorough attenuating AGE formation and neuroinflammatory responses induced by MGO-AGEs in vitro.

## 1. Introduction

Advanced glycation end products (AGEs) or glycotoxins are products of the Maillard reaction between reducing sugars and amino acid residues in several proteins and nucleic acids, through glycation, autoxidation, or lipoxidation [1,2]. AGEs can either be produced endogenously or are present in processed foods, which undergo a browning reaction (for instance, the barbequing of red meat produces excessive AGEs, making it a major source of exogenous AGEs) [1]. Exogenous and endogenous AGEs are well-known mediators of several human ailments, including cardiovascular, hepatic, renal, and neurological complications. In particular, diabetes can lead to the formation of endogenous AGEs in the body [3]. Hyperglycemia-mediated autoxidation normally results from the production of major AGE precursors, namely, methylglyoxal (MGO) and glyoxal (GO) [4]. The high reactivities of MGO and GO quicken their reaction with amino acids (specifically arginine, lysine, and cysteine), resulting in the formation of AGEs [5]. AGEs derived from these precursors are responsible for several organ toxicities [6]. MGO and GO accumulation and subsequent AGE formation is inhibited by the glyoxalase (GLO I and II) system, which induces the breakdown of MGO and GO to D-lactate and glycolic acid, respectively [7]. Despite various reports on MGO- and GO-AGE-mediated toxicity in different organs, their roles in cells of the central nervous system are yet to be reported.

Accumulating evidence has revealed that AGEs are associated with aging-related disorders, such as neurological complications, skin photoaging, psoriasis, metabolic disorders, diabetes, and vascular complications [8,9,10,11,12,13]. Previous studies mainly focused on the harmful roles of AGE in diabetes, as it is associated with abnormal glucose metabolism and AGE accumulation. In fact, diabetes-associated AGEs can have direct neurodegenerative roles, as diabetes can also result in different neurological complications. The major mechanisms through which diabetes may influence neuroinflammation include glucose toxicity, AGE formation, AGE receptor (RAGE) activation, cerebrovascular injury, and vascular inflammation [14]. AGE–RAGE interactions in brain cells (specifically microglia) and infiltrating macrophages lead to their activation towards the inflammatory phenotype, resulting in excessive production of proinflammatory mediators, a major cause of neuroinflammation and neurodegeneration [15]. Complications mediated by such AGE may be controlled by either inhibiting AGE formation, promoting AGE breakdown, or preventing AGE–RAGE interactions. 

In this study, we propose sulforaphane (SFN), a representative Nuclear factor erythroid 2-related factor 2 (Nrf-2)-activating phytochemical [16], as a potential candidate against inflammatory cascades induced by MGO-derived AGE. SFN, a natural isothiocyanate, is mostly present in cruciferous vegetables as a glucoraphanin conversion product. It is known to have antioxidant, anti-inflammatory, anti-apoptotic, cytoprotective, and anti-diabetic effects [17,18]. The aqueous extract of glucoraphanin-rich broccoli sprouts inhibits AGE formation and AGE-induced inflammatory reactions in endothelial cells and rat aorta [19,20]. Furthermore, SFN-mediated inhibition of AGE–RAGE interaction is known to be responsible for pericyte protection against AGE-induced toxicity [21]. However, its role against MGO-AGEs-mediated neurotoxicity in neuronal cells or the nervous system is yet to be revealed. In previous studies, we reported the ability of SFN-enriched broccoli and SFN alone to significantly downregulate lipopolysaccharide (LPS)-mediated inflammatory cascades [22,23]. These independent previous studies indicated that sulforaphane is effective in inhibiting the MGO-AGEs-mediated neuroinflammatory reaction. In this study, we investigated whether SFN can be a potential candidate for ameliorating microglial activation mediated by MGO-AGEs and the corresponding neuroinflammatory events. 

## 2. Materials and Methods

### 2.1. Materials

Fetal bovine serum, penicillin–streptomycin (PS), Dulbecco’s modified Eagle’s medium, and 3-(4,5-dimethylthiazol-2-yl)-2,5-diphenyltetrazolium bromide (MTT) powder were purchased from Invitrogen (Carlsbad, CA, USA). LPS, MGO, and SFN were procured from Sigma Aldrich (St. Louis, MO, USA). Competitive enzyme-linked immunosorbent assay (ELISA) kits of tumor necrosis factor α (TNF-α) and Interleukin 6 (IL-6) were purchased from R&D Systems (Minneapolis, MN, USA). Primary antibodies for cyclooxygenase2 (COX-2), β-actin, and Glyceraldehyde 3-phosphate dehydrogenase (GAPDH) were purchased from Santa Cruz Biotechnology (Dallas, TX, USA), whereas inducible nitric oxide synthase (iNOS) was ordered from Abcam (Cambridge, UK). Other primary antibodies such as α-tubulin, p-IκB, Nuclear factor κB (NF-κB), p38, c-Jun N terminal kinase (JNK), Extracellular signal-regulated kinase (ERK), Glycogen synthase kinase 3 beta (GSK3β), pJNK, pp38, pERK, pGSK3β, NLR Family Pyrin Domain Containing 3 (NLRP3), and IκB were purchased from Cell Signaling Technology (Beverly, MA, USA). Protein lysis buffer (Pro-Prep) was obtained from iNtRON Biotechnology (Seongnam-si, Gyeonggi-do, Korea).

### 2.2. Preparation of AGEs

MGO-modified BSA-AGEs (MGO-AGEs) were prepared by incubating 5 mg/mL BSA and 10 mM MGO in the presence of 0.02% sodium azide (pH 7.4) at 37 °C for 8 weeks, as described previously [24]. Formation of AGEs was characterized or quantified by two different methods: measurement of fluorescent intensity and by performing ELISA. Small molecular weight (7 kDa) cut-off membranes were used to dialyze the AGEs for better filtration and prevention of errors with particulate matter. The filtered solution was evaporated/freeze-dried, and the fine powders of AGE were obtained. AGE formation was analyzed and characterized by two methods. The first method was by measuring the fluorescence intensity at 355/460 nm wavelength for excitation/emission in a VICTOR X3 microplate reader (Perkin Elmer, Waltham, MA, USA). The second method was through measuring the AGE-BSA competitive ELISA according to the kit protocol. 

### 2.3. AGEs Competitive ELISA Assay

AGEs characterization was performed by using AGEs competitive ELISA assay. We followed the protocol of OxiSelect Advanced Glycation End Product (AGE) competitive ELISA kit from Cells biolabs (Cat No: STA-817, San Diego, CA, USA) with slight modification. AGE level in cells following MGO-AGEs and with or without SFN treatment was also measured by using this technique. BV2 cells (1.5 × 10^6^ cells per well) were seeded onto a six-well plate and pretreated with SFN, and then stimulated with MGO-AGE for 30 min. After 24 h incubation, the cells were lysed with PRO-PREPTM protein extraction solution for 24 h, and cell lysates were centrifuged at 12,000× *g* for 20 min at 4 °C. To evaluate the effect of SFN on AGEs levels, an AGEs competitive ELISA kit assay was performed in accordance with the manufacturer’s protocol.

### 2.4. AGEs Formation Inhibition Assay

To determine the direct effect of SFN on AGEs formation, we performed an assay as described by Kiho et al. [25] with minor changes. BSA (5 mg/mL) was homogenized with 10 mM MGO or GO in phosphate-buffered saline (PBS) (pH 7.4), followed by the addition of SFN (400 μM). Sodium azide (0.02%) was then added, and the mixture was incubated at 37 °C for 7 days. Thus, altered AGE level was determined to assess the effects of SFN on the inhibition of AGEs formation. Aminoguanidine (AGD; 1 mM) was used as a positive control. The amount of AGEs formed was evaluated by measuring the fluorescence intensity at 355/460 nm (excitation/emission) using a VICTOR X3 plate reader (Perkin Elmer, Waltham, MA, USA).

### 2.5. AGE Breakdown Activity Assay

The 2,4,6-trinitrobenzene sulfonic acid (TNBSA) assay was performed to verify the effect of SFN on AGE breakdown, as described previously [26] with minor changes. Briefly, 1 mL of MGO-BSA was homogenized with either SFN or the positive control (AGD; 1 mM) and incubated for 24 h. At the following day, TNBSA (0.1 %) and NaHCO_3_ (4 %) were added, and the mixture was incubated at 37 °C for 2 h. The reaction was stopped by adding 10% SDS and 1 N HCl. AGEs breakdown products were analyzed by measuring the absorbance at 540 nm using microplate reader (Molecular Devices, San Jose, CA, USA). 

### 2.6. Nitrite Production and Cell Viability Assays

BV2 microglial cells, originally developed at the University of Perugia (Perugia, Italy) by Dr. V. Bocchini were kindly provided by Dr. E. Choi from Korea University, Seoul, South Korea. BV2 cells were maintained in Dulbecco’s Modified Eagle Medium (DMEM) and stored in a humidifier incubator maintaining 37 °C and 5% CO_2_. BV2 cells (4 × 10^4^ cells per well) were seeded onto a 96-well plate and incubated overnight. Cells were treated with either vehicle (control) or different doses of SFN (dissolved in DMSO with a final concentration of 0.05%) 30 min prior to MGO-AGEs stimulation and incubated for an additional 24 h. Nitrite production was measured by Griess reagent assay, as described previously [27]. Conditioned medium from treated cells was mixed with an equal volume of Griess reagent in a new 96-well plate for the measurement of nitrite production, as described previously [28,29]. Cells were then processed for cell viability assay, as determined previously [29,30]. Briefly, cells were incubated with 0.5 mg/mL MTT solution for approximately 1 h in the dark. Once the live cell stain turned blue, we removed the MTT solution and the cells were then exposed to dimethyl sulfoxide (DMSO), which dissolved the purple formazan color (representative of live cells). This color change was quantified by measuring the absorbance at 570 nm using a microplate reader (Molecular Devices, San Jose, CA, USA).

### 2.7. LDH Production Assay

To determine the cytotoxicity of the AGEs, we performed lactate dehydrogenase (LDH) assay. LDH assay kit was obtained from Invitrogen. LDH production was determined as described previously [31]. Briefly, BV2 cells were treated with different concentrations of MGO-AGE and were incubated for 24 h. Conditioned medium (50 μL) from the treated cells were collected and mixed with equal amounts of LDH mixture (LDH substrate and buffer). This resulted in a color change into a brown-red color because of the conversion of lactate to pyruvate in the presence of LDH enzyme, which was evaluated my measuring the absorbance at 450 nm. This experiment was performed exactly as described by LDH assay kit protocol from Thermo Fisher Scientific/Invitrogen (catalogue no. C20300, Waltham, MA, USA).

### 2.8. ROS Production Assay

Intracellular oxidative stress induced by the treatment of MGO-AGEs was measured by evaluating the increased level of reactive oxygen species (ROS) production by performing 2′,7′-dichlorofluroscein diacetate (DCF-DA) staining in the BV2 microglial cells. Cells were pretreated with SFN 30 min prior to MGO-AGEs stimulation and incubated for 1 h. DCF-DA staining/ROS production assay were performed as described previously [32] with slight modifications. Briefly, treated BV2 cells were washed with PBS and incubated with 10 µM of 2′,7′-dichlorofluroscein diacetate (DCF-DA) for 20 min and incubated in a light protected covering in an incubator maintaining 37 °C and 5% CO_2_. After completion of staining time, cells were washed with PBS and fluorescent cells were counted by using flow cytometer.

### 2.9. NF-κB Assay (Nuclear and Cytosolic Extraction)

BV2 cells were treated with SFN and then stimulated with MGO-AGEs for 1 h. The cytosolic and nuclear fraction was separated using a Nuclear/Cytosolic Extraction Kit (Active Motif, Carlsbad, CA, USA) following the manufacturer’s instructions. IκB and pIκB cytosolic fraction proteins and NF-κB nuclear fraction proteins were then processed for Western blot analysis. 

### 2.10. Western Blot Analysis

Protein expression was analyzed by Western blotting analysis, as described previously [33]. BV2 cells (1.5 × 10^6^ cells per well) were seeded onto a six-well plate and pretreated with SFN in the presence or absence of AGEs and incubated for different time points. Cells were washed with phosphate-buffered saline (PBS) and lysed using Pro-Prep lysis buffer. To evaluate the phosphorylated forms of mitogen-activated protein kinase (MAPKs) (pERK, pp38, pJNK) and pGSK3β, we supplemented Pro-Prep lysis buffer with a phosphatase inhibitor-like cocktail. Cell lysates were separated, and the protein content was estimated using Bradford’s assay. Protein samples (30 μg) were separated by sodium dodecyl sulfate polyacrylamide gel electrophoresis (SDS-PAGE), transferred to nitrocellulose membranes, and incubated overnight with primary antibodies for p-IκB, NF-κB, p38, JNK, ERK, GSK3β, pJNK, pp38, pERK, pGSK3β, NLRP3, IκB (all 1:1000; Cell Signaling), and α-tubulin. The membranes were then washed and incubated with respective secondary antibodies. Protein bands were visualized using Enhanced chemiluminescence (ECL) reagent (Amersham Pharmacia Biotech, Little Chalfont, UK). The protein band intensity was quantified by the Image Master 2D Elite software (version 3.1; Amersham Pharmacia Biotech, Little Chalfont, UK).

### 2.11. ELISA

BV2 cells were treated either with AGEs only or SFN pretreatment 30 min prior to AGEs treatment, and then incubated for 24 h. Conditioned medium from the treated cells was collected and processed for ELISA to determine the secreted levels of proinflammatory cytokines (TNF-α and IL-6) according to the manufacturer’s protocol (R&D Systems, Minneapolis, MN, USA).

### 2.12. Statistical Analysis

Results are expressed as mean ± standard error of the mean (SEM). One-way analysis of variance followed by Tukey post-hoc test was used to measure statistical significance with GraphPad Prism 5 (La Jolla, CA, USA). *p* < 0.05 was considered statistically significant. 

## 3. Results

### 3.1. SFN Pretreatment Lowered MGO-AGEs Formation and Attenuated the Production of Neuroinflammatory Mediators Induced by MGO-AGE in BV2 Microglial Cells

**Figure 1:**
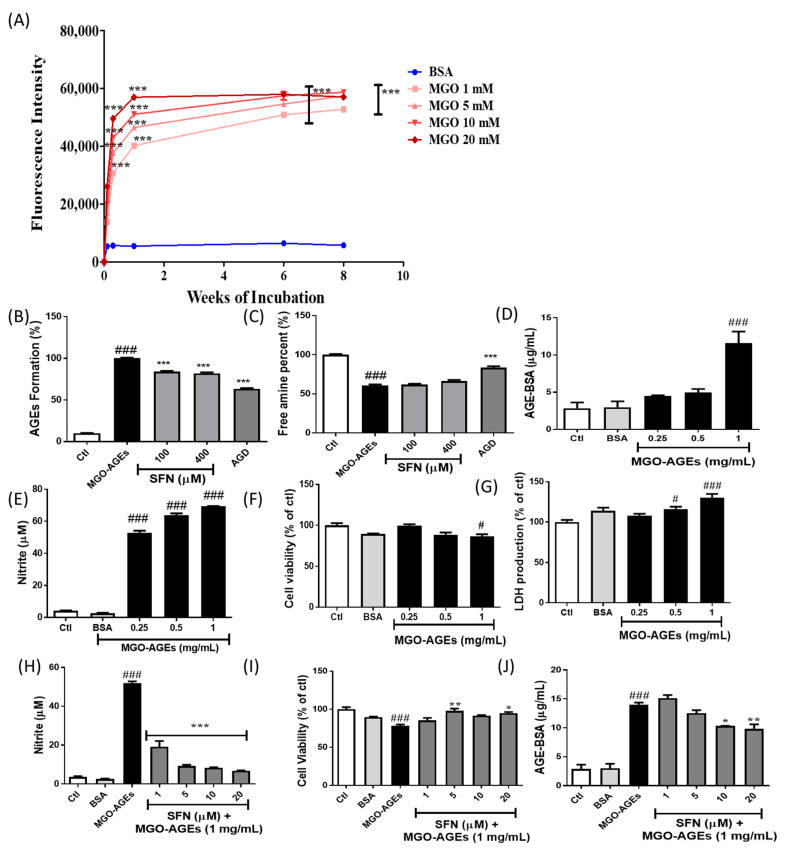
Sulforaphane (SFN) pretreatment reduced the methylglyoxal (MGO)-advanced glycation end product (AGE)-mediated inflammatory cascades in microglial cells. BSA was incubated with MGO (1, 5, 10, and 20 mM) for 8 weeks and formation of MGO-AGEs was determined in a concentration- and time-dependent manner (**A**). SFN was incubated with AGE to determine its effects on AGE formation and breakdown (**B**,**C**). AGE level was measured by using AGE-competitive ELISA assay (**D**). BV2 cells were treated with various concentrations of MGO-AGEs to determine nitrite production (**E**), cell viability (**F**), and lactate dehydrogenase (LDH) production (**G**). BV2 cells were pretreated with SFN at 30 min prior to MGO-AGEs (1 mg/mL) stimulation and incubated for 24 h to determine effects of SFN on nitrite production (**H**), cell viability (**I**), and AGEs level by AGE-competitive ELISA assay (**J**). * *p* < 0.05, ** *p* < 0.01, *** *p* < 0.001 indicates significant differences compared with AGE alone, whereas # *p* < 0.05 and ### *p* < 0.001 indicate significant differences compared with an untreated control group.

MGO was used to prepare AGEs through incubation with BSA for 8 weeks. This led to increased AGE production, as evidenced by a significant increase in fluorescence intensity at early time points. A marked increase in AGE formation after up to 6 weeks of incubation was followed by plateau/saturation from the eighth week (Figure 1A). As SFN was proposed as a potential candidate against MGO-AGEs, we investigated the role of SFN in MGO-AGEs formation and breakdown. Incubation of MGO-AGEs with SFN (100 and 400 µM) and AGD (1 mM), a positive control, significantly lowered MGO-AGEs formation (Figure 1B). However, SFN was not effective in inducing AGE breakdown, as evidenced by free amine production (Figure 1C). Only AGD showed the ability to significantly induce MGO-AGEs breakdown (Figure 1C). These data clearly indicated that SFN was mainly responsible for the prevention of MGO-AGEs formation rather than breakdown. Amount of AGE in the prepared MGO-AGEs powder was also characterized by using AGE-competitive ELISA. A total of 1 mg/mL of MGO-AGEs was enough to produce a significant amount of AGEs in the cell lysate measured by ELISA assay (Figure 1D). However, SFN was unable to alter formation and breakdown of GO-AGEs (Figure S1A–C).

In addition, we examined the role of MGO-AGEs in microglial activation. MGO-AGEs, at 0.25, 0.5, and 1 mg/mL, dramatically induced nitrite production and cytotoxicity in BV2 microglial cells (Figure 1E,F). Supportively, MGO-AGEs significantly increased the LDH production in BV2 cells at the concentrations of 0.5 and 1 mg/mL (Figure 1G), suggesting its toxicity to microglial cells possibly through its activation or inflammation. MGO-AGEs-induced nitrite production was significantly attenuated upon SFN treatment (Figure 1H) without cell toxicity (Figure 1I), suggesting that SFN could antagonize MGO-AGEs-induced oxidative stress in activated microglia, which was also observed with GO-AGEs (Figure S1D–G). We also performed an AGEs-competitive ELISA to determine the level of intracellular AGE in in BV2 cells following SFN and MGO-AGE treatment. A dramatically high amount of intracellular AGEs was observed in the MGO-AGEs (1 mg/mL)-treated cell lysate, however, SFN treatment, especially 10 and 20 μM, significantly lowered the level of AGE inside the cells (Figure 1J).

### 3.2. SFN Pretreatment Lowered Reactive Oxygen Species (ROS) Production Induced by MGO-AGEs in Microglial Cells

**Figure 2:**
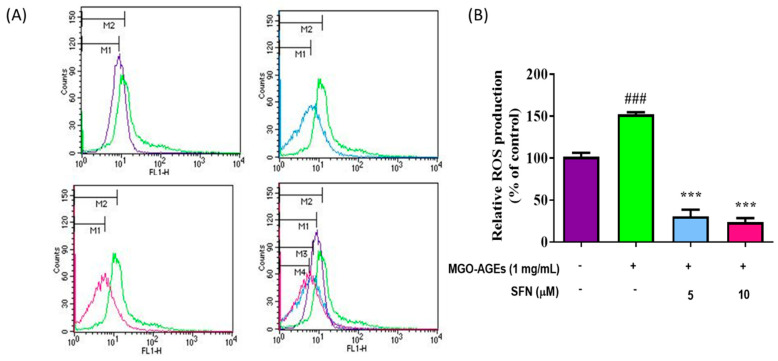
SFN pretreatment reduced MGO-AGEs-induced reactive oxygen species (ROS) production in microglial cells. BV2 cells were pretreated with SFN following MGO-AGEs (1 mg/mL) treatment. ROS production in the MGO-AGEs group and MGO-AGEs + SFN co-treatment groups was evaluated using flow cytometry and compared with that in the control group (**A**,**B**). *** *p* < 0.001 indicates significant differences compared with AGEs alone, whereas ### *p* < 0.001 indicates significant differences compared with an untreated control group.

In addition to increasing nitrite production, MGO-AGEs treatment also significantly increased ROS production in microglial cells. However, this significant increase in ROS production was dramatically reversed upon SFN treatment (Figure 2A,B), suggesting that SFN can attenuate MGO-AGEs-mediated ROS production in BV2 microglial cells. 

### 3.3. SFN Pretreatment Attenuated the Expression of Neuroinflammatory Proteins and MAPK Effector Signaling

**Figure 3:**
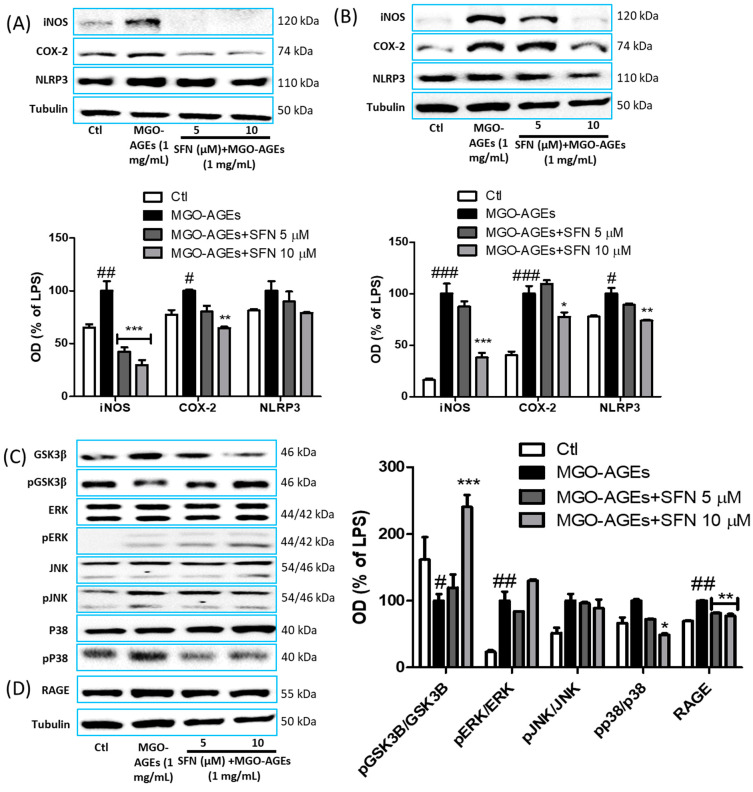
SFN pretreatment reduced the protein expression of inflammatory mediators in MGO-AGEs-stimulated microglial cells. BV2 cells were pretreated with SFN followed by MGO-AGEs (1 mg/mL) treatment. Protein expression and densitometric analysis results of iNOS, COX-2, and NLRP3 at 6 and 24 h after MGO-AGEs treatment (**A**,**B**). Protein expression and densitometric analysis results of MAPKs and GSK3β (**C**) at 30 min after MGO-AGEs treatment, and expression of AGE receptor (RAGE) was determined at 24 h after MGO-AGEs treatment (**D**). * *p* < 0.05, ** *p* < 0.01, and *** *p* < 0.001 indicate significant differences compared with AGE alone, whereas # *p* < 0.05, ## *p* < 0.01, and ### *p* < 0.001 indicate significant differences compared with an untreated control group.

After confirming the inhibitory role of SFN in nitrite production, we determined the effects of SFN on iNOS and COX-2 protein expression in MGO-AGEs-activated microglia. SFN pretreatment dramatically attenuated the expression of the inflammatory protein iNOS and COX-2 expression at 6 h after MGO-AGEs stimulation (Figure 3A). Activated microglia-mediated NLRP3 is considered to be the major inflammasome responsible for the detrimental roles of the inflammatory microglial phenotype; hence, this effect was also evaluated following SFN pretreatment and MGO-AGE activation. However, at this time point, SFN treatment did not inhibit NLRP3 expression (Figure 3A). Nevertheless, at 24 h after MGO-AGEs stimulation, SFN significantly inhibited NLRP3 expression in MGO-AGEs-activated microglia, along with iNOS and COX-2 (Figure 3B), suggesting that SFN could attenuate the inflammatory responses of activated microglia. In addition, SFN pretreatment decreased GSK3β activation and p38 phosphorylation, but the alteration of ERK and JNK phosphorylation was not significant (Figure 3C). These effects of SFN were also observed with GO-AGEs (Figure S1L–O).

Receptor of AGE (RAGE) is the major receptor responsible for proinflammatory responses of AGE [34]. Therefore, we also determined whether SFN-mediated anti-inflammatory responses in AGE-activated microglia were related with attenuated RAGE expression. Indeed, SFN treatment decreased the expression of RAGE (Figure 3D) at 24 h after AGEs stimulation, suggesting that SFN could inhibit the AGE–RAGE axis, which may be responsible for attenuating AGE–RAGE interaction, leading to the decreased downstream inflammatory cascades in activated microglia.

### 3.4. SFN Pretreatment Reduced NF-κB Translocation and Proinflammatory Cytokine Production in MGO-AGEs-Activated Microglial Cells

**Figure 4:**
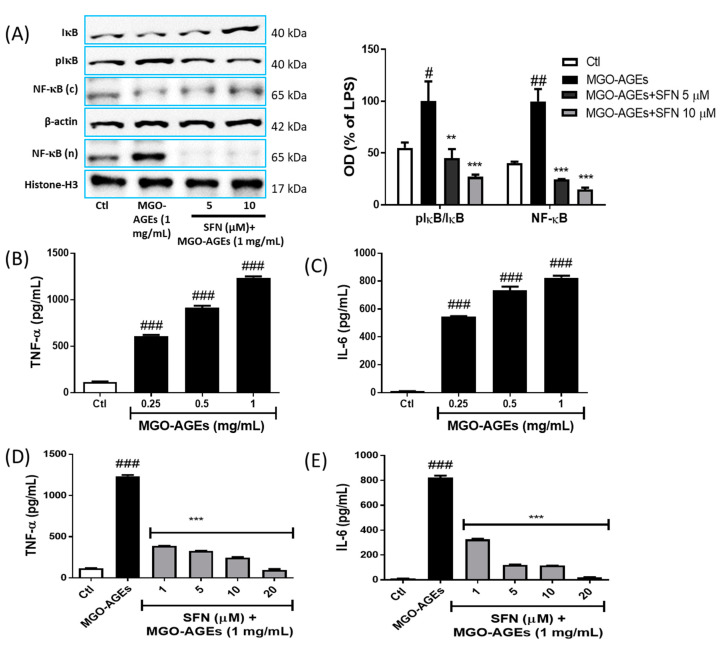
SFN pretreatment reduced MGO-AGEs-mediated NF-κB translocation and proinflammatory cytokine secretion in microglial cells. BV2 cells were pretreated with SFN followed by MGO-AGEs (1 mg/mL) treatment (1 h for NF-κB and 24 h for proinflammatory cytokines). Cytosolic (c) and nuclear (n) protein expression and densitometric analysis results of NF-κB, IκB, and pIκB (**A**). Secreted levels of TNF-α and IL-6 in BV2 cells following treatment with different concentrations of MGO-AGEs (**B**,**C**). Secreted levels of TNF-α and IL-6 in BV2 cells pretreated with SFN and treated with MGO-AGEs (**D**,**E**). ** *p* < 0.01 and *** *p* < 0.001 indicate significant differences compared with AGE alone, whereas # *p* < 0.05, ## *p* < 0.01, and ### *p* < 0.001 indicate significant differences compared with an untreated control group.

We further evaluated the effect of SFN on MGO-AGEs-mediated NF-κB activation/translocation 1 h [35] after MGO-AGEs treatment. MGO-AGEs-driven NF-κB translocation to the nucleus was inhibited by SFN pretreatment (Figure 4A), which was further supported by increased cytosolic NF-κB expression. Moreover, reduced IκB degradation, as evidenced by reduced IκB phosphorylation, was observed in the SFN-treated group. Moreover, MGO-AGEs-mediated IκB degradation (by its phosphorylation) leads to NF-κB translocation to the nucleus; this sequence of events was reversed by SFN treatment. Inhibition of these NF-κB transcriptional pathways subsequently inhibits the production of proinflammatory cytokines. In this study, MGO-AGEs exposure significantly increased the production of proinflammatory cytokines, TNF-α, and IL-6 (Figure 4B,C), which was decreased upon SFN treatment (Figure 4D,E). SFN also attenuated the production of proinflammatory cytokines in GO-AGEs-primed BV2 cells (Figure S1H–K). Altogether, our results suggested that SFN possessed a strong potency to lower the AGE-mediated inflammatory responses in activated microglia.

## 4. Discussion

AGE are reported to be major pathological factors in several human ailments, and, lately, research has focused on the resolution of AGE-mediated complications [36]. Accumulation of AGEs precursors has been reported in diabetic complications, and therefore AGEs accumulation may be the cause of diabetic toxicities, including neurotoxicity, as characterized by neuropathy and other neuronal complications such as Alzheimer’s disease, multiple sclerosis (MS), Parkinson’s disease, and stroke [37,38]. Moreover, increased MGO levels may actively participate in AGE formation. However, previous studies investigating AGE responsible for toxicity mostly focused on BSA-AGEs prepared with sucrose or fructose and rarely on MGO-AGEs [39]. High levels of glucose-based AGE are present in processed foods, including MGO-AGEs, which are the major breakdown products of glucose and are responsible for AGE formation. Hence, appropriately addressing the effects of MGO-AGEs can help raise awareness regarding protection against possible exposure to or toxicity of endogenous or dietary AGE [40]. MGO-mediated AGE formed in glycolysis-driven cells are reported to be actively involved in MS lesions [13]. Nevertheless, the exact role of MGO-AGEs and the inflammatory cascades caused by MGO-AGEs, specifically in neuroinflammation, remain unknown. On the one hand, AGE were previously reported to play proinflammatory roles; inflammation is a major cause of central nervous system (CNS) disorders [41]. On the other hand, RAGE activation through the AGE–RAGE interaction can cause damage to the blood–brain barrier (BBB), increasing the permeability of the BBB to toxic substances, which can result in neuroinflammation and neurodegeneration [42]. However, MGO-mediated toxicity and neuroinflammation-like conditions have not been reported experimentally. In this study, we explored the toxicities of MGO-AGEs and proposed SFN as a protective agent against the corresponding MGO-AGEs-mediated inflammatory complications via regulation of the inflammatory microglial phenotype [43]. AGEs and LPS can activate RAGE and toll like receptor 4 (TLR4) receptors, respectively, to exert their inflammatory effects through downstream signaling. The activation of RAGE and TLR4 in microglia or macrophage share common inflammatory pathways, indicating their role in neuroinflammation [27,34,44,45,46]. SFN showed a strong potency to inhibit microglial activation to the inflammatory phenotype and to increase the levels of anti-inflammatory phenotypic markers in LPS-activated microglia [22,23]. We therefore believed that SFN exerted similar pharmacological effects in MGO-AGEs-stimulated microglial cells to that in LPS-stimulated microglial cells. 

Given that AGE-mediated RAGE activation is a well-known pathway for macrophage/microglia activation to its inflammatory phenotype [15] and most of the RAGE activators can lead to the numerous downstream signaling pathways related to production of inflammatory in microglia [47,48], we focused on the role of MGO-AGEs and their ability to alter inflammatory microglial biomarkers [49,50]. We observed that MGO-AGEs treatment sharply increased NO and proinflammatory cytokine production, resulting in significant cytotoxicity to microglial cells. Similarly, increased expression of iNOS, COX-2, NLRP3, MAPKs, nuclear NF-κB, and RAGE in microglial cells was observed following MGO-AGEs treatment. These results were supported by an independent previous study by Chen et al., who observed that AGE can mediate the RAGE/Rho/ Rho-associated protein kinase (ROCK) pathway, thereby upregulating microglial biomarkers such as iNOS, COX-2, and NLRP3 by upregulating NF-κB translocation [15]. Free NF-κB can be translocated to the nucleus, resulting in increased transcription of inflammatory mediators [51,52]. This could be a possible reason for IκB degradation due to increased pIκB and upregulated nuclear NF-κB, which ultimately increased the production of inflammatory cytokines and other mediators after MGO-AGEs treatment. SFN significantly lowered these cascades, indicating that SFN is a strong candidate against AGEs-mediated toxicity. In addition, previous studies clearly reported the cross-talk between Nrf-2 and NF-κB, as Nrf-2 negatively regulates the NF-κB-mediated proinflammatory cascades [53]. It is evident that SFN, being the strong regulator of Nrf-2 activation, results in the inhibition of NF-κB signaling.

The significant increase in MGO-AGE-induced microglial RAGE expression was attenuated by SFN, suggesting SFN-mediated RAGE inhibition as the primary step for lowering AGE–RAGE-mediated downstream inflammatory events. This result supported previous findings that SFN inhibited AGE–RAGE expression and downregulated the inflammatory cascades in pericytes and endothelial cells [20,21]. Apart from those of MGO-AGEs, SFN treatment dramatically decreased the levels of inflammatory mediators, which were increased by GO-AGEs. These results suggested the high potency of SFN to lower neuroinflammation induced by various types of AGEs. MGO-AGEs were recently implicated in the pathogenesis of MS [13]. Our present results supported that the additional involvement of MGO-AGEs-mediated neuroinflammatory cascades may be a major cause of neurological complications such as MS. These results revealed a target physiological pathway that can be controlled by SFN to lower inflammatory cascades in activated microglia; however, the mechanism underlying the reduction in MGO-AGEs formation remains unclear. The glyoxalase system is the key to detoxify MGO-AGEs toxicity; hence, increased glyoxalase I/II and glutathione may help decrease AGE-mediated toxicity [7]. SFN decreased MGO-AGEs formation as well as its interaction with RAGE, and inhibited AGE-induced oxidative stress/inflammation by decreasing ROS production in microglia, possibly via the redox biological activation of the cellular antioxidant system, Nrf-2/ Heme oxygenase-1 (HO-1), as previously described [23,54,55]. Despite identifying SFN as an appealing phytochemical that can counter AGE-mediated neuroinflammation, we have a few limitations to report. In this study, we prepared AGEs on our own in the laboratory. Preparation of AGEs using MGO, GO, or sucrose/fructose is relatively easy but the amount of AGEs formed and the complex it contains might vary between the batches, which might result in an alteration in the amount of desired AGEs or its extent of toxicity or biological effects. Next, we cannot assert whether the concentration of AGEs we used in this study is pathologically relevant or not. More research on the appropriate method for the preparation and characterizations of AGEs are needed in order to be able to explore their role in human health and disease in terms of future independent studies. In addition, in terms of the role of MGO-AGEs on other CNS cell types following in vivo experiments, it is tempting to explore their exact role in the pathophysiological system, which possibly may help in terms of the prevention or treatment of neuroinflammatory disorders.

## 5. Conclusions

In this study, MGO, a highly reactive glycolytic product, was shown to actively participate in AGE formation. It clearly increased the oxidative stress and inflammatory cascades through microglial activation towards the inflammatory phenotype, leading to neuroinflammation and subsequent neurodegeneration. Interestingly, the neuroinflammatory cascades induced by MGO-AGEs were ameliorated by SFN treatment through downregulation of oxidative stress and inflammatory pathway activation, reduction in AGE–RAGE interaction, and reduction in AGE formation. Therefore, SFN may be a strong candidate against neuroinflammation induced by MGO-AGEs or neurodegenerative diseases caused by chronic glycative stress. 

## Supplementary Materials

The following are available online at https://www.mdpi.com/2076-3921/9/9/792/s1, Figure S1: SFN treatment modulated GO-AGE-mediated inflammatory events in microglial cells.
