# Supplementary material for: Sulforaphane Inhibits MGO-AGE-Mediated Neuroinflammation by Suppressing NF-κB, MAPK, and AGE–RAGE Signaling Pathways in Microglial Cells
Source: Antioxidants (Basel). 2020 Aug 26;9(9):792. doi: 10.3390/antiox9090792 (PMC7554773; doi:10.3390/antiox9090792)

Supplementary Materials: *Supplementary Figure 1. SFN treatment modulated GO-AGE-mediated inflammatory events in microglial cells.* (A) Formation of GO-AGE. ***p <0.001 indicates significant differences compared with BSA. Effects of SFN exposure on the formation (B) and breakdown (C) of GO-AGE. GO-AGE (1 mg/mL) stimulation increased nitrite production (D) and decreased cell viability (E), which were attenuated upon SFN treatment (F, G). GO-AGE stimulation increased production of proinflammatory cytokines TNF-α and IL-6 (H, I) in microglial culture, which was attenuated upon SFN treatment (J, K). SFN attenuated GO-AGE mediated iNOS, COX-2, and NLRP3 expression in microglial cells in 6 h (L) and 24 h (M) after GO-AGE stimulation. SFN treatment decreased the GO-AGE-mediated MAPKs and GSK3β expression in microglial cells (N, O). ***p <0.001 indicates significant differences compared with GO-AGE, whereas ##p <0.01 and ###p <0.001 indicates significant differences compared with a control group.

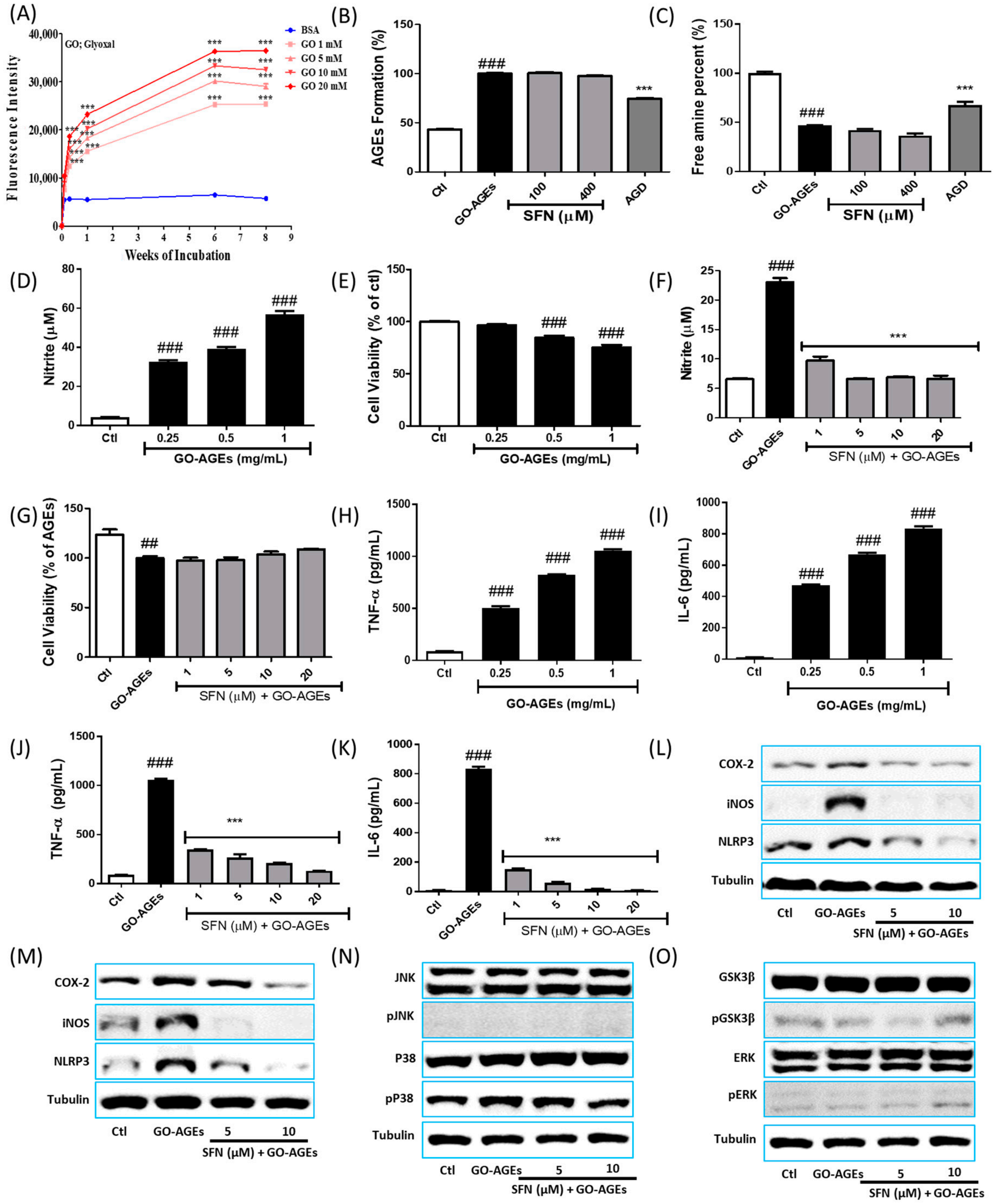

Supplement: Supplementary file 1 [file antioxidants-09-00792-s001.pdf]
